# Contrast-induced encephalopathy mimicking stroke after a second cerebral DSA: an unusual case report

**DOI:** 10.1186/s12883-021-02457-5

**Published:** 2021-11-05

**Authors:** Jiaying Li, Guanshu Qi, Huani Zhang, Gang Chen, Shuting Wang, Minli Yan, Zhichao Sun

**Affiliations:** 1 The First Clinical Medical College of Zhejiang Chinese Medical University, Hangzhou, 310053 China; 2 Department of Radiology, The First Affiliated Hospital of Zhejiang Chinese Medical University (Zhejiang Provincial Hospital of Traditional Chinese Medicine), Hangzhou, 310006, China; 3 Department of Neurology, The First Affiliated Hospital of Zhejiang Chinese Medical University (Zhejiang Provincial Hospital of Traditional Chinese Medicine), Hangzhou, 310006 China

**Keywords:** Cerebral angiography, Contrast media, Encephalopathy, Stroke

## Abstract

**Background:**

Contrast-induced encephalopathy (CIE) is a rare complication of the angiography process. CIE may mimic stroke symptoms clinically and subarachnoid hemorrhage radiologically. Previous CIE cases occurred after the initial digital subtraction angiography (DSA) scan. Here, we encountered an unusual case of CIE mimicking a stroke with an internal carotid artery (ICA) aneurysm and ipsilateral ICA stenosis that occurred after a second DSA procedure.

**Case presentation:**

A 77-year-old female with a history of hypertension and coronary heart disease underwent two cerebral DSA procedures over 1 week. She was given the same nonionic and iso-osmolar Visipaque agent (smaller than 200 ml) for both procedures. However, neurological complications only occurred after the second DSA procedure. On the first diagnostic cerebral DSA, she was diagnosed with an intracranial aneurysm of the left ICA with moderate stenosis (approximately 50%) in the initial part of the ipsilateral ICA. However, after the second aneurysm embolization procedure by DSA, she developed right hemiplegia, aphasia, and epilepsy, mimicking left middle cerebral artery occlusion. An emergency CT showed a diffuse hyperdensity in the left subarachnoid space, mimicking SAH. MRI demonstrated that the lesion was hyperintense on T2WI, FLAIR imaging, and DWI but was normal on ADC mapping. On postoperative Day 6, her neurologic deficits had completely resolved after initial fluid restriction, corticosteroid treatment, and rehydration.

**Conclusion:**

This case indicates that clinicians should consider the occurrence of CIE following any angiography procedure, even if the initial cerebral DSA procedure is successful and without complications.

## Background

Contrast-induced encephalopathy (CIE) is a rare neurological complication of diagnostic and therapeutic cerebral angiography. The incidence of CIE patients is 0.06% for those undergoing CAG, 0.3–1% for those undergoing vertebral angioplasty, and 2.9% for those undergoing endovascular coil treatment of posterior circulation aneurysm [1–4]. CIE may mimic a stroke clinically and subarachnoid hemorrhage (SAH) radiologically, creating a diagnostic dilemma. Previous CIE case reports occurred after the initial cerebral digital subtraction angiography (DSA) within 12 h of the procedure [5]. Most patients recover completely within 48–72 h without any neurological impairment, but there are rare cases of irreversible or even fatal CIE [4–6]. Here, we encountered an unusual and unreported CIE case with internal carotid artery (ICA) aneurysm and ipsilateral ICA stenosis that occurred after the second cerebral DSA procedure using a nonionic and iso-osmolar contrast agent, not the first DSA.

## Case presentation

A 77-year-old female presented with slurred speech, facial weakness, and left limb numbness and was admitted into our hospital. The patient had a history of hypertension and coronary heart disease, without diabetes or smoking. The patient’s hepatic function, renal function, coagulable state, and other laboratory tests were normal. Brain MRI without contrast revealed a subacute cerebral infarction near the right side of the lateral ventricle. The patient was given supportive treatment and improved. Nonetheless, an intracranial aneurysm of the left ICA was incidentally found on cranial MR angiography (MRA). Afterward, this patient underwent a diagnostic cerebral angiography and an additional therapeutic cerebral angiography.

After the second therapeutic angiography, the patient presented with right hemiplegia and aphasia when she was resuscitated from anesthesia. Neurological examination revealed mixed aphasia, left eye gaze, reduced pupillary light reflex, loss of right muscle strength (level 0/5), a positive right Babinski sign, and lack of response to painful stimuli. At first, we suspected stroke due to the clinical findings which mimicked a left middle cerebral artery (MCA) occlusion. According to the TOAST criteria, the “stroke” symptom could be classified as large artery atherosclerosis [7]. However, the patient’s clinical findings did not match the imaging results which showed a patent left MCA. Therefore, CIE was suggested. The patient was given 200 mg methylprednisolone QD IV, 124 mL of 20% mannitol Q8H and rehydration fluids after emergent CTA and MRI examinations. 54 h later, the patient suddenly developed a generalized tonic-clonic seizure lasting about half a minute. She achieved remission after symptomatic treatment. 72 h later, the patient’s right hemiplegia and aphasia began to improve, as the muscle strength of the right lower limb increased to 3/5 and the right upper muscle strength remained at 0–1/5. Finally, after fluid restriction dehydration, short-term corticosteroid treatment, and rehydration resuscitation, all neurologic deficits achieved complete remission on a postoperative Day 6.

The first diagnostic cerebral DSA procedure was conducted to further confirm the existence of the left intracranial ICA saccular aneurysm with a size of 6.9 × 6.0 mm, with a total of 150 mL nonionic, iso-osmolar Iodixanol (Visipaque, 320 mg I/ml, Bayer Healthcare, USA) IV. It also showed extracranial moderate stenosis (approximately 50%) in the initial segment of the left ICA. The internal carotid artery and vertebral artery angiography showed the anterior, middle, and posterior cerebral arteries. One week later, the patient underwent a second angiographic treatment for aneurysm embolization under general anesthesia. On this second procedure, the 6F intracranial support catheter (Navien, RFX072–105-08MP, EV3, America) was guided over the stenotic left ICA to the extracranial distal segment of ICA (the level of first cervical vertebra) without local artery vasospasm, stenosis, or the stasis of the contrast agent. A total of 160 mL Iodixanol was injected during the second therapeutic DSA.

**Fig. 1:**
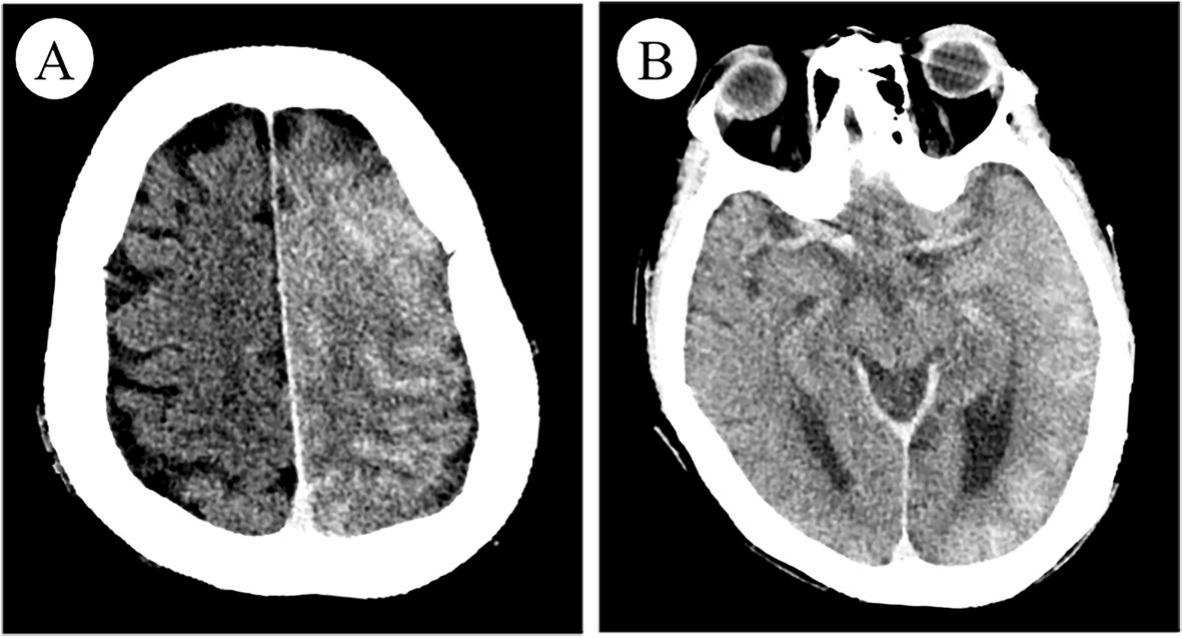
Emergent brain CT angiography (CTA). Axial brain CTA showing the left cerebral hemisphere swelling and diffuse high-density in the subarachnoid space

**Fig. 2:**
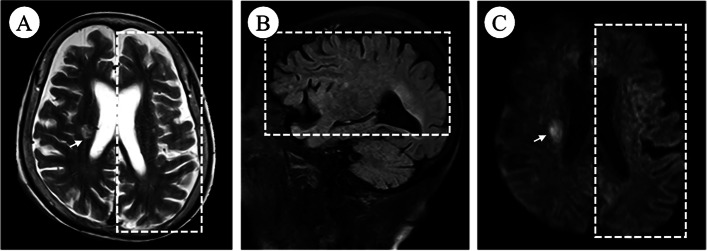
Brain MRI after 4 hours. Brain MRI showed normal signal on axial T2WI (**A**) and sagittal FLAIR (**B**), but mild gyral hyperintense on axial DWI (**C**) in the left brain (dotted square); subacute cerebral infarction near the right lateral ventricle (white arrow with tail)

**Fig. 3:**
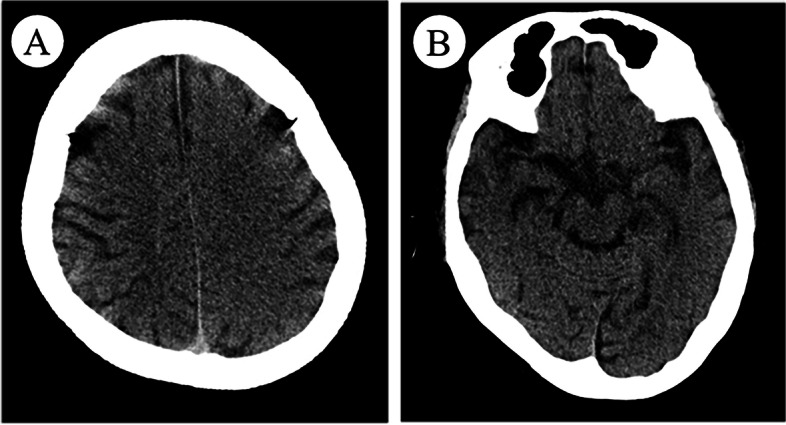
Repeat brain CT after twenty hours. Axial brain CT showed the resolution of left cerebral hemisphere swelling and diffuse high-density

**Fig. 4:**
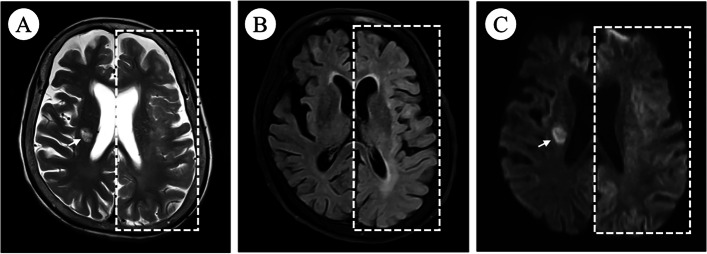
Repeat brain MRI after twenty-eight hours. Repeat brain MRI showed mild gyral hyperintense on axial T2WI (**A**), FLAIR (**B**), and DWI (**C**) in the left brain (dotted square); subacute cerebral infarction near the right lateral ventricle (white arrow with tail)

After the second interventional DSA procedure, an emergent head CT angiography (CTA) scan showed the left cerebral hemisphere swelling, shallowing of the sulci, and diffuse high-density in the subarachnoid space without any cerebral artery occlusion or stenosis (Fig. 1). 4 h later, brain MRI showed diffuse swelling in the left cortex with a normal signal on T2-weighted imaging (T2WI), fluid-attenuated inversion recovery (FLAIR), and mild hyperintense on diffusion-weighted imaging (DWI) **(**Fig. 2**)**. 20 h later, repeat brain CT showed the resolution of left cerebral hemisphere swelling and diffuse high-density **(**Fig. 3**)**. However, 28 h later, repeat non-contrasted brain MRI showed mild gyral hyperintense in the left hemisphere on T2WI, FLAIR imaging, and DWI with a normal ADC mapping **(**Fig. 4**)**. There were no indications of acute cerebral infarction.

## Discussion and conclusions

Contrast-induced encephalopathy (CIE) is a rare and self-limited complication associated with angiography procedures. The neurologic manifestations of CIE can mimic stroke, as shown in this case. This patient presented with right hemiplegia, aphasia, and seizure after a cerebral interventional procedure. The clinical syndrome mimicked a left MCA occlusion, according to the TOAST criteria, which could be classified as large-artery atherosclerosis. However, the patient’s clinical findings did not match the imaging results. CIE was suggested as the main cause of this presentation. The patient required 6 days to recover from the neurological deficits with fluid restriction to reduce intracranial pressure, short-term corticosteroid treatment, and subsequent fluid resuscitation.

At the present, the mechanism of CIE is not clear. The widely accepted mechanism suggests that the contrast media disrupts the blood-brain barrier (BBB) and penetrates the interstitial space, leading to direct neuronal toxicity and brain edema [8, 9]. In this case, age, chronic hypertension, and coronary heart disease may be the risk factors that lead to BBB and cerebral automatic regulation impairments. In theory, a higher-osmotic and ionic contrast concentration may induce the contraction of endothelial cells and lead to greater permeability. Previous cases reported that all types of contrast agents regardless of their permeability or ionic states can induce CIE [10, 11], even if the iso-osmotic contrast agent was used such as in this case.

The first diagnostic cerebral DSA confirmed the presence of the left ICA intracranial saccular aneurysm and extracranial moderate stenosis. It was suggested that resolving the ipsilateral ICA stenosis first may lead to aneurysm rupture due to the sudden increase in cerebral blood flow [12]. The patient also showed no symptoms of left ICA stenosis. Thus, 1 week later, she underwent the second therapeutic DSA procedure after which the neurological complications occurred. She was given the same iso-osmolar contrast agent and a large volume of contrast (smaller than recommended for the prevention of toxicity 200 mL) [3]. Yet CIE occurred following the second DSA. This finding is likely the result of the contrast agent distributing into the ipsilateral cerebral hemisphere during subsequent treatment. Although the unresolved moderate stenosis of ipsilateral ICA may be related to CIE, the abnormal imaging manifestations occurred on the left hemisphere, including the occipital lobe supplied by the left posterior cerebral artery. This contradicted imaging findings which suggested a patent PCA.

It is important to combine clinical symptoms with imaging manifestations to diagnose CIE. The imaging manifestation of extravasation of contrast media can mimic SAH on CT imaging, including the brain edema, hyperdensity of the subarachnoid space, cortical and subcortical local enhancement [1–11]. CIE should be differentiated from acute cerebral infarction. DWI and ADC sequences can quantify water diffusion to make a distinction between CIE and acute cerebral infarction. Acute cerebral infarction is described as diffusion-limited due to cytotoxic edema, which behaves as hyperintense on DWI and hypointense on ADC. In contrast, CIE can usually exhibit gyral hyperintense on FLAIR and T2WI, with or without high signal on DWI, but ADC mapping is normal.

CIE may overlap with atypical posterior reversible encephalopathy syndrome (PRES) between clinical and neuroradiological features. But the brain imaging of PRES reveals symmetric abnormalities predominantly involving the parieto-occipital regions of both cerebral hemispheres, which presents as hyperintense on T2WI and FLAIR imaging due to vasogenic edema [13, 14]. In this case, imaging showed abnormal signals in a unilateral brain hemisphere including frontal, temporal, parietal and occipital lobes. DSA and postoperative CTA showed no cerebral vascular vasospasm and stenosis. Thus, the clinical and radiological findings were consistent with CIE in our case.

In conclusion, although the first cerebral DSA procedure was successful and without complications, clinicians should still consider the occurrence of CIE following any angiography procedure. In patients with risk factors and internal carotid artery stenosis, even more attention should be given to the diagnosis of CIE. In the future, additional studies will be needed to explore whether internal carotid artery stenosis, the number of cerebral angiographies, and the interval time of angiography are risk factors for CIE.

## Data Availability

Not applicable.

## Abbreviations

ADC: Apparent diffusion coefficient
BBB: Blood-brain barrier
CIE: Contrast-induced encephalopathy
CTA: Computer tomography angiography
DSA: Digital subtraction angiography
DWI: Diffusion-weighted imaging
ICA: Internal carotid artery
MCA: Middle cerebral artery
MRI: Magnetic resonance imaging
PRES: posterior reversible encephalopathy syndrome
SAH: Subarachnoid hemorrhage
